# Incidence and Determinants of Ventilation Tubes in Denmark

**DOI:** 10.1371/journal.pone.0165657

**Published:** 2016-11-22

**Authors:** Tine Marie Pedersen, Anna-Rosa Cecilie Mora-Jensen, Johannes Waage, Hans Bisgaard, Jakob Stokholm

**Affiliations:** 1 COPSAC, Copenhagen Prospective Studies on Asthma in Childhood, Herlev and Gentofte Hospital, University of Copenhagen, Copenhagen, Denmark; 2 Department of Pediatrics, Naestved Hospital, Naestved; Denmark; Universite Paris Descartes, FRANCE

## Abstract

**Background and objectives:**

Many children are treated for recurrent acute otitis media and middle ear effusion with ventilation tubes (VT). The objectives are to describe the incidence of VT in Denmark during 1997–2011 from national register data, furthermore, to analyze the determinants for VT in the Copenhagen Prospective Studies on Asthma in Childhood_2010_ (COPSAC_2010_) birth cohort.

**Methods:**

The incidence of VT in all children under 16 years from 1997–2011 were calculated in the Danish national registries. Determinants of VT were studied in the COPSAC_2010_ birth cohort of 700 children.

**Results:**

Nationwide the prevalence of VT was 24% in children aged 0 to 3 three years, with a significant increase over the study period. For all children 0–15 years, the incidence of VT was 35/1,000. In the VT population, 57% was male and 43% females. In the COPSAC_2010_ birth cohort, the prevalence of VT during the first 3 years of life was 29%. Determinants of VT were: maternal history of middle ear disease; aHR 2.07, 95% CI [1.45–2.96] and siblings history of middle ear disease; aHR 3.02, [2.11–4.32]. Paternal history of middle ear disease, presence of older siblings in the home and diagnosis of persistent wheeze were significant in the univariate analysis but the association did not persist after adjustment.

**Conclusion:**

The incidence of VT is still increasing in the youngest age group in Denmark, demonstrating the highest incidence recorded in the world. Family history of middle ear disease and older siblings are the main determinants for VT.

## Introduction

Otitis media is a common infection in early childhood and a common cause of children’s health care utilization, partly from ventilation tube (VT) insertion [1]. Denmark did not have an official guideline for ventilation tube insertions until 2015. Now the indications are similar to the US guideline that recommend VT when middle ear effusion is persistent for over 3 months and accompanied by either documented hearing loss (>20 dB) or speech, language, learning, balance problems, ear discomfort, or reduced quality of life among others [2,3]. Recurrent acute otitis media is defined as either a minimum of three episodes of acute otitis media (AOM) in a six months period or more than three episodes in a year [4,5]. VT insertion is not recommended for children with recurrent AOM without middle ear effusion [2]. Benefits of VT must be balanced against the associated risk of complications including the general anesthesia [6–8].

A meta-analysis on risk factors for otitis media has shown that upper airway tract infections, low social status and attending daycare increases the risk of otitis media, and also a borderline significant association between increased otitis media and both male sex and living with siblings [9]. To our knowledge no former analysis of determinants of VT insertions have been performed. Previous studies have focused on risk factors for otitis media.

The rate of VT insertions varies greatly across developed countries, and does not directly reflect socioeconomic affluence [10–16]. The incidence is under 10/1000 in United Kingdom, Canada and United States but over 30/1000 in Iceland and Denmark, S1 Fig.

The aim of this study is to analyze the rate of VT insertions in Denmark over time and compare with other countries. Furthermore, we will utilize data from the Copenhagen Prospective Studies on Asthma in Childhood_2010_ (COPSAC_2010_) birth cohort [17] to analyze determinants for VT in Danish children.

## Methods

This is a 2-step study design where we use Danish registry data to calculate and describe the development of the incidence of ventilation tube insertions. Furthermore, we use data from the COPSAC_2010_ cohort study to analyze determinants of ventilation tubes.

### Ethics

The registry-based study was performed on existing data in national registries and was approved by the Danish Data Protection Agency (J.no. 2012-41-0388). Since subjects were not contacted as part of the study, written informed consent was not required.The COPSAC_2010_ cohort study was performed according to the principles of the Declaration of Helsinki and was approved by the Ethics Committee of Copenhagen (H-B-2008-093) and the Danish Data Protection Agency (2008-41-2599), and written informed consent was obtained from all families.

### Study Population

#### Registry data

The study includes the total number of Danish children who had VT between 1^st^ January 1997 and 31^st^ December 2011 in either hospital setting or primary care. The National Patient Registry contains data on all hospital contacts linked with a unique personal identification number (assigned by the Danish Civil Registration System to all people with permanent residency in Denmark), dates of admission and discharge and diagnoses at discharge classified according to the international classification of diseases (ICD-10) [18,19]. Cases of VT insertions were identified as children below 16 years with the ICD-10 diagnosis: KDCA20. Information regarding procedures performed in the private Ear-Nose-Throat (ENT) surgeon clinics was obtained from The National Health Insurance Service Registry [20]. The VT procedure codes extracted were 3009 and 3109, but to calculate the number of children having VT only the code for the first ear was used (3009).

To calculate the incidence of ventilation tubes, we used the number of children in Denmark each year from 1997–2011.

#### COPSAC_2010_ cohort

COPSAC_2010_ is a birth cohort of 700 children [17]. Mothers were recruited during pregnancy and children were included at one week of age and followed prospectively in our research unit for thorough clinical phenotyping. The children have currently been followed by the study pediatricians with scheduled visits at 1 week, 1, 3, 6, 12, 18, 24, 30, 36, 48, 60 months.

#### Determinants of ventilation tube insertion

Information regarding maternal age at birth, maternal smoking during pregnancy, maternal asthma, delivery method, gestational age, birth weight, sex and older children in the household was obtained by personal interview at the 1 week visit in the COPSAC clinic. Older siblings include biological siblings and half siblings, with primary address in the home of the child. Days solely breastfed and age at beginning of daycare were obtained longitudinally from clinical interviews during the first year of life. Information regarding the household income was obtained at the 2-year visit in the clinic and data from the child’s 1-year birthday until 2 years was used to avoid the year the mother had been on maternity leave resulting in a lower income than normally. Information regarding the family members’ history of middle ear disease was obtained from interviews at age 3 years. Information obtained after the 1-week visit was not acquired for all 700 children. The numbers included in the analysis are shown in Table 1.

**Table 1:** Determinants of ventilation tubes.

	Participants	Tubes	No Tubes	*P* value	Adjusted HR (95% CI)
All	700	205	495		
***Maternal-related characteristics***	*** ***				
Maternal age at birth, mean years (range)	698	32.3 (21.5–48.3)	32.2 (19.1–44.0)	0.9261	-
Maternal smoking during pregnancy, % (n)	696	8% (16)	8% (38)	0.9539	-
Maternal asthma, % (n)	697	27% (55)	26% (129)	0.7897	-
Household income *	677			0.7333	-
- Low % (n)	56	10% (19)	8% (37)		-
- Medium % (n)	366	54% (108)	54% (258)		-
- High % (n)	255	37% (73)	38% (182)		-
***Child-related characteristics***	*** ***				
Cesarean section, 151 children,% (n)	700	22% (46)	21% (105)	0.7195	-
Mean number of days exclusively breastfed, mean (range)	692	103.4 (0–255)	103.0 (0–266)	0.8660	-
Mean GA weeks mean (range)	700	39.7 (33.0–42.3)	39.9 (29.4–42.3)	0.1429	-
Mean birth weight kg, mean (range)	700	3.5 (1.9–5.2)	3.5 (1.3–5.0)	0.9797	-
Sex, boys, % (n)	700	56% (111)	49%(249)	0.1117	-
Mean days at beginning of daycare (range)	688	320 (156–946)	334 (180–1154)	0.0577	-
Older siblings present, %(n)	700	64%(132)	53% (264)	**0.0072**	1.34 (0.86; 2.05)
Season of birth, % (n)	700			0.2768	-
Winter	215	26% (55)	32% (160)		-
Spring	186	26% (52)	27% (134)		-
Summer	149	22% (46)	21% (103)		-
Fall	150	25% (52)	20% (98)		-
Child with persistent wheeze anytime before the age of 3 years	662	25% (48)	17% (79)	**0.0191**	1.18 (0.79; 1.76)
***Family history of middle ear disease*:**	*** ***	*** ***	*** ***	*** ***	
- Paternal % (n)	615	22% (38)	11% (48)	**0.0002**	1.48 (0.97; 2.27)
- Maternal % (n)	643	34% (62)	17% (79)	**<0.0001**	2.07 (1.45; 2.96)
- Siblings % (n)	518	59% (90)	25% (92)	**<0.0001**	3.02 (2.11; 4.32)

* Household income at age 2: Low (Below 50.000 Euro), Medium (50.000–110.000), High (Above 110.000)

#### Genetic risk score

The otitis media gene risk score was based on 21 polymorphic genetic variants found to be associated with recurrent otitis media in childhood in a large questionnaire based genome-wide association study (n = 121,810) [21]. The variants were weighted according to their odds ratios, and risk alleles were summed and z-score transformed.

#### Genotyping of the COPSAC_2010_ cohort

DNA was purified from blood cells from the children and multiple single-nucleotide polymorphisms were genotyped genome-wide using the high throughput Illumina HumanOmniExpressExome bead chip platform (Illumina, Inc., San Diego, CA, USA). Genotyping was performed at AROS Applied Biotechnology AS, Aarhus, Denmark. Genotyping quality control included removal of gender mismatches, duplicates, ethnic outliers, and Hardy-Weinberg equilibrium (p>10^−6^) outliers. A genotyping call rate of at least 95% was required. Correct familial relations were verified by Mendel error rates and identity-by-descent analyses. All quality control was performed using PLINK software [22,23]. The HumanOmniExpressExome chip was imputed to the 1000 genomes phase 3 imputation panels (CEU individuals) using Mach 1.0, Markov Chain Haplotyping, and IMPUTE2.

#### Clinical predictors

A disease diary was filled out daily by the parents from birth to 3 years of age, and validated by the research pediatricians during clinical visits, including clinical symptoms or medical treatments in three categories: lung symptoms, infections and eczema. We required completed diaries during >90% of the period to be included in the analysis. The lung symptom category have been used to capture children with recurrent wheeze episodes and who have been treated with inhaled corticosteroids according to a predefined algorithm [24]. These children were diagnosed with persistent wheeze. AOM episodes and likewise persistent middle ear effusion are indications for VT, see causality diagram S2 Fig. These factors were not used in the analysis regarding determinants of VT but to describe disease prevalence in the cohort. All AOM episodes from birth to 3 years of age were captured in the infection category of the diary.

To diagnose middle ear effusion we used a flat curve at tympanometry measurement. Children were examined at age one, two and three years with a tympanometry (MT10, Interacoustics, Denmark).

### Statistics

#### Registry data

Data from The National Patient Registry and The National Health Insurance Service Registry was combined to evaluate the total number of VT insertions in Denmark from 1997–2011. We described the development over time in the number of VT procedures and calculated the difference between the numbers of procedures in hospitals compared to private ENT-clinics. Changes over time were analyzed by linear regression analysis.

#### COPSAC_2010_ data

Univariate associations between predictors and ventilation tubes ever (0–3 years) were examined. Associations between categorical variables and VT insertions were analyzed by chi-squared tests or Fisher’s exact test where expected counts in any cells were less than 5. Household income was analyzed with a Cochran-Armitage trend test. Normal distributed continuous variables were tested with t-test. Variables not normally distributed were analyzed using Wilcoxon rank-sum tests. Univariate significant determinants of VT insertions were thereafter analyzed in Cox proportional hazards regression models to adjust for all other significant determinants. All adjusted results were expressed as hazard ratios (HR) with 95% confidence intervals (CI). A significance level of 5% was used in all analysis. Missing data were not imputed. R version 3.2.3 [25] was used for calculating genetic risk scores. SAS version 9.4 for Windows (SAS Institute Inc., Cary, NC, US) was used for all other analyses.

## Results

### Registry data

The total number of person years for children 0–15 years of age from 1997–2011 was 15.593.538. The average number of children living in Denmark was 1,039,569 in the study period. The number of children <16 years having VT from 1997–2011 was 288,224 and several of the children received VT more than once, which made the total number 514,575 of VT insertions. The average number of VT insertions was 36,196 annually.

**Fig 1:**
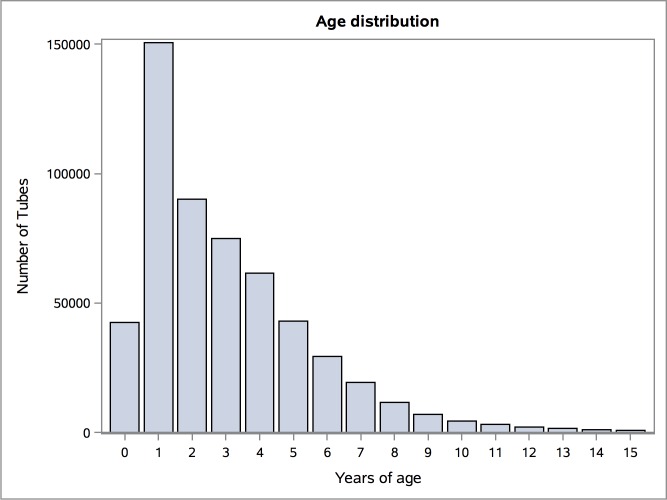
Age distribution of children 0–15 years who received ventilation tubes between 1997–2011 in Denmark.

**Fig 2:**
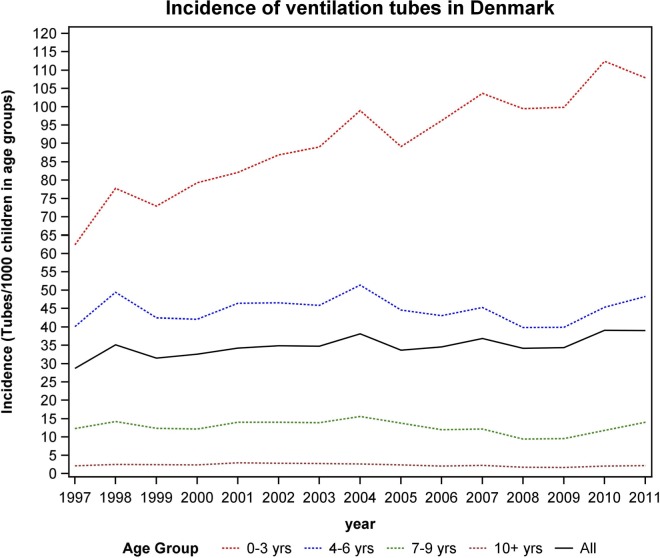
Incidence of ventilation tubes in Denmark. The incidence (ventilation tubes/1000 children 0–3 years of age) from 1997–2011 in Denmark.

**Fig 3:**
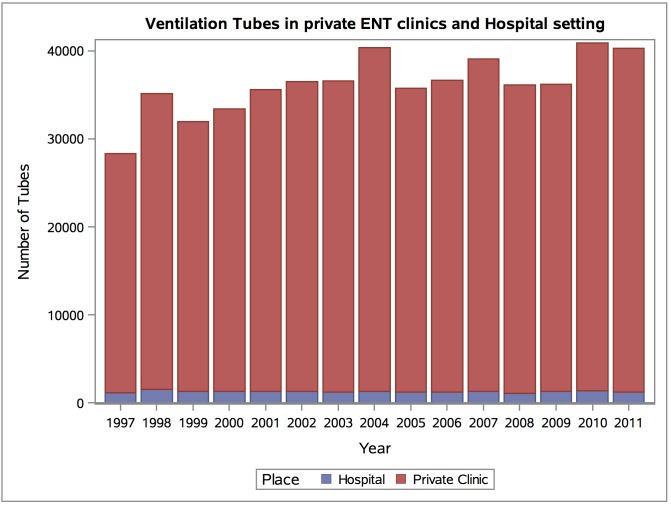
Number of ventilation tubes placed in private ENT-clinics compared to hospitals.

Most children have VT insertions in early childhood (Fig 1). The national prevalence of VT in the first 3 years of life was 24% in children born in 2009. For all Danish children under 16 years, the incidence was 35/1,000. During the observation period, there has been an overall increase in VT insertions (Fig 2). Especially in the children aged 0–3 years the number of VT insertions increased from 62/1,000 to 108/1,000, 43% increase, corresponding to an increased by 2.94 per year, SE 0.27, p<0.0001. There was a sex difference. Boys more often received VT compared to girls, respectively 57% and 43%. Among children having VT insertions, 53% received tubes one time, 24% two times, 11% three times and 12% of the children had VT more than 3 times. The hospitals account for 4% of the procedures and the private ENT-clinics for 96% (Fig 3).

### COPSAC_2010_ cohort

The mean age of the first VT insertion was 16 months (SD 4.9). The youngest child was 5.8 months old. The accumulated prevalence of VT was 5% (n = 37) after one year, 22% (n = 151) after 2 years, and 29% (n = 205) after 3 years, very similar to the national prevalence of VT.

AOM episodes in the first 3 years of life among children in the cohort were captured in the daily disease diary; 74% (N = 515) of the cohort had information of AOM episodes for >90% of the first 3 years of life. Of these, 67% (n = 346) had at least one AOM episode from age 0–3 years.

Flat tympanometry curves was found in 52% (292/563) at the 1 year clinic visit, 37% (221/595) at the age of 2 years, and 27% (n = 163/454) at the 3 years visit. In total, 66% (n = 454/614) of the children had middle ear effusion at least once before the age of 3.

### Determinants of ventilation tube insertion

A main determinant for VT insertion was family history of middle ear disease. Maternal history persisted in the adjusted analysis; HR 2.07, [1.45–2.96] and likewise history of middle ear disease among siblings; HR 3.02, [2.11–4.32] (Table 1). Older children in the home were also associated with a higher risk of VT; OR 1.58, 95% CI, [1.13–2.21], p = 0.0072, but not in the adjusted analysis. A diagnosis of persistent wheeze was also associated with a significant higher risk of VT insertions, OR 1.62, 95% CI, [1.08–2.43], p = 0.0191, but the association did not persist in the adjusted analysis. On average the children who received VT before the age of 3 had started 14 days earlier in daycare compared to children without VT, p = 0.0577. There was no association between sex and VT in the COPSAC cohort and no associations were found between VT and maternal age, maternal smoking during pregnancy, household income, maternal asthma, delivery by cesarean section, length of exclusive breast feeding, gestational age, birth weight, season of birth.

Using a genetic risk score, we did not observe a significant association between this risk score and time to receiving VT before the age of 3; HR 0.97, 95% CI [0.84–1.12], p = 0.68.

## Discussion

### Main findings

The annual incidence in children during the observation period 1997–2011 is to our knowledge the highest incidence reported from any country and without obvious reasons. Children in the COPSAC_2010_ birth cohort had a period prevalence of VT of 29% before the age of 3, comparable to the national prevalence of 24% in the same period. Determinants for VT insertion were AOM, family history of middle ear disease and older children in the home.

### Strengths and limitations

The Danish registries are unique because they contain information regarding all procedures and contacts with the health care system linked to personal identification numbers for the complete population. Thereby all VT insertions in Denmark, both procedures performed in the hospital setting and in ENT-clinics could be included in the analysis. The registries do not contain any information of the VT indication, so we were not able to evaluate whether all children fulfilled the criteria for VT insertion.

The COPSAC_2010_ birth cohort has been followed prospectively from birth and the collected data on the 700 children has been linked to the registry data for information on all VT procedures. This is a robust outcome compared to former studies that have used either recurrent AOM or middle ear effusion with more varied and unclear definitions.

The COPSAC_2010_ data contains information of several exposures in early life and prospective disease diaries which provide information on all AOM episodes during the first 3 years of life. It is a unique dataset because of the difficulty in getting accurate information regarding small children’s diseases. In addition, we obtained annual tympanometry measurement, though this is limited by the nature of a spot measurement, and children may have experienced middle ear effusion between the examinations. However; we captured middle ear effusion among a high percentage of the children. As the majority of children experience middle ear effusion during their first three years of life, this could almost be considered a natural phenomenon. We observed a difference between sex regarding VT and probably non significant because of lack of power. This is a limitation, but the sex difference for otitis media has been described in other studies [9].

### Interpretation

The incidence of VT in Denmark is the highest compared to available data from other developed countries [10–15,26] (S1 Fig) and with a continuing increase over the study period (Fig 2).

In the United States 6.8% of children have had VT before the age of 3 associated with their insurance status and access to treatment [2]. In the United Kingdom otitis media prevalence decreased after the introduction of the Pneumococcal Conjugated Vaccine (PCV) vaccine in the national vaccine program [27]. PCV vaccine was introduced in 2007 in Denmark yet the VT rate has only been increasing since (Fig 2).

We would expect the Danish population to be comparable to especially the other Nordic countries with similar disease prevalence. Still, very different incidences of VT insertions are observed (S1 Fig). The Danish population has free access to ENT specialists like in Island and they have a similar incidence of VT. [28,29]. In Sweden, the general practitioner is gate keeper with referral being needed to be examined by an ENT surgeon and have a 3–4 fold lower incidence of VT [30].

Children receive VT for either middle ear effusion or recurrent AOM. 67% of all the COPSAC children had had at least one AOM episode before the age of three and AOM was associated with a significantly increased risk of VT. We found that middle ear effusion was present at one, two and three years of age among 51%, 37% and 27% respectively. We found that 66% of the children had middle ear effusion at minimum one of the annual measurements, but this was not found significantly associated with VT. Middle ear effusion could be considered a natural phenomenon [31] with a high prevalence in early childhood and is not alone an indication for VT insertion. Middle ear effusion can cause impaired hearing and ventilation tubes leads to a small hearing improvement [32], however, the overall beneficial effects of VT are debated. A randomized controlled trial concluded no long term beneficial effects of ventilation tubes on language, speech or cognitive development [33–35], though children with middle ear effusion and a delayed speech development might benefit from VT insertion [32,36–38]. Frequent infections and sleep problems because of otitis media may result in reduced quality of life for the child and the whole family, but the effect of VT on this is ambiguous [39,40].

Family history of middle ear disease was among the most dominant factors predicting VT insertion. It has been shown that otitis media is 40–70% heritable and recently genome-wide association study studies have been published on genetic variants influencing the proneness of otitis media [41,42]. We did not find any association between the genetic risk score and receiving VT in the COPSAC_2010_ cohort before the age of 3. This can be explained by phenotypic differences (the genetic risk score is done from a questionnaire based study, both an inherent strength and weakness) or lack of power. The genetic risk score phenotype is based on those suffering from recurrent AOM may therefore represent differences compared to risk of VT as children often receive VT for persistent middle ear effusion.

Intriguingly, this may also be indicative of the fact that many children in the COPSAC_2010_ cohort (as well as in Denmark) receive VT for other reasons than otitis media; e.g. decreased sleep quality, delayed speech development or decreased quality of life.

Other determinants of VT included older children in the home and early start in daycare. These factors are both accompanied by increased exposure to pathogens and infections early in life. In Denmark, children are typically attending daycare centers within the first year of life. Our study is consistent with findings in previous literature. A study from the United States also found that daycare attendance was a risk factor of VT [43], and a recent meta-analysis analyzing risk factors of otitis media found association to both siblings and attending daycare [9].

Children diagnosed with persistent wheeze had a higher rate of VT insertions. The association between recurrent upper airway infections and middle ear effusion have been shown in other studies [9,44], and it is probably due to a higher propensity for pathogen colonization in the airways in general in these children [45].

It has previously been shown that males suffer from more AOM than girls [46]. This was confirmed in the data from the national registries with a ration of approx. 3:2.

Children in the COPSAC_2010_ cohort had a slightly higher prevalence of VT insertion, 29% compared to 24% in the national registers among children born in the same year as the COPSAC_2010_ cohort. This higher prevalence could be explained by the close follow-up of the COPSAC_2010_, where children with middle ear effusion at the yearly tympanometry measurements were recommended to get an appointment at an ENT-clinic.

## Conclusion

In Denmark, we have the highest incidence of VT insertions reported worldwide with a continuing increase. This suggests that health-care practices rather than evidence-based guidelines determine the use of VT. Heritability, children with persistent wheeze and older siblings in the home significantly influence the risk of VT. The determinants of VT are comparable to previous studies on either AOM or middle ear effusion, but this is the first study using VT as outcome.

## Supporting Information

S1 Fig The incidence of ventilation tubes in Denmark compared to other countries. The overall incidence of ventilation tubes in Denmark of 35/1000 for children 0–15 years of age compared to incidences in other developed countries published in the literature [10–15,28,29].
(PDF)

S2 Fig Causality diagram. (PDF)

## Abbreviations

VT: Ventilation Tubes
AOM: Acute Otitis Media
COPSAC_2010_: COpenhagen Prospective Study on Asthma in Childhood_2010_
ENT: Ear-Nose-Throa
HR: Hazard Rati
CI: Confidence interval
SE: Standard Erro
PCV: Pneumococcal Conjugated Vaccine
