# Supplementary figures and images for: Incidence and Determinants of Ventilation Tubes in Denmark
Source: PLoS One. 2016 Nov 22;11(11):e0165657. doi: 10.1371/journal.pone.0165657 (PMC5119727; doi:10.1371/journal.pone.0165657)

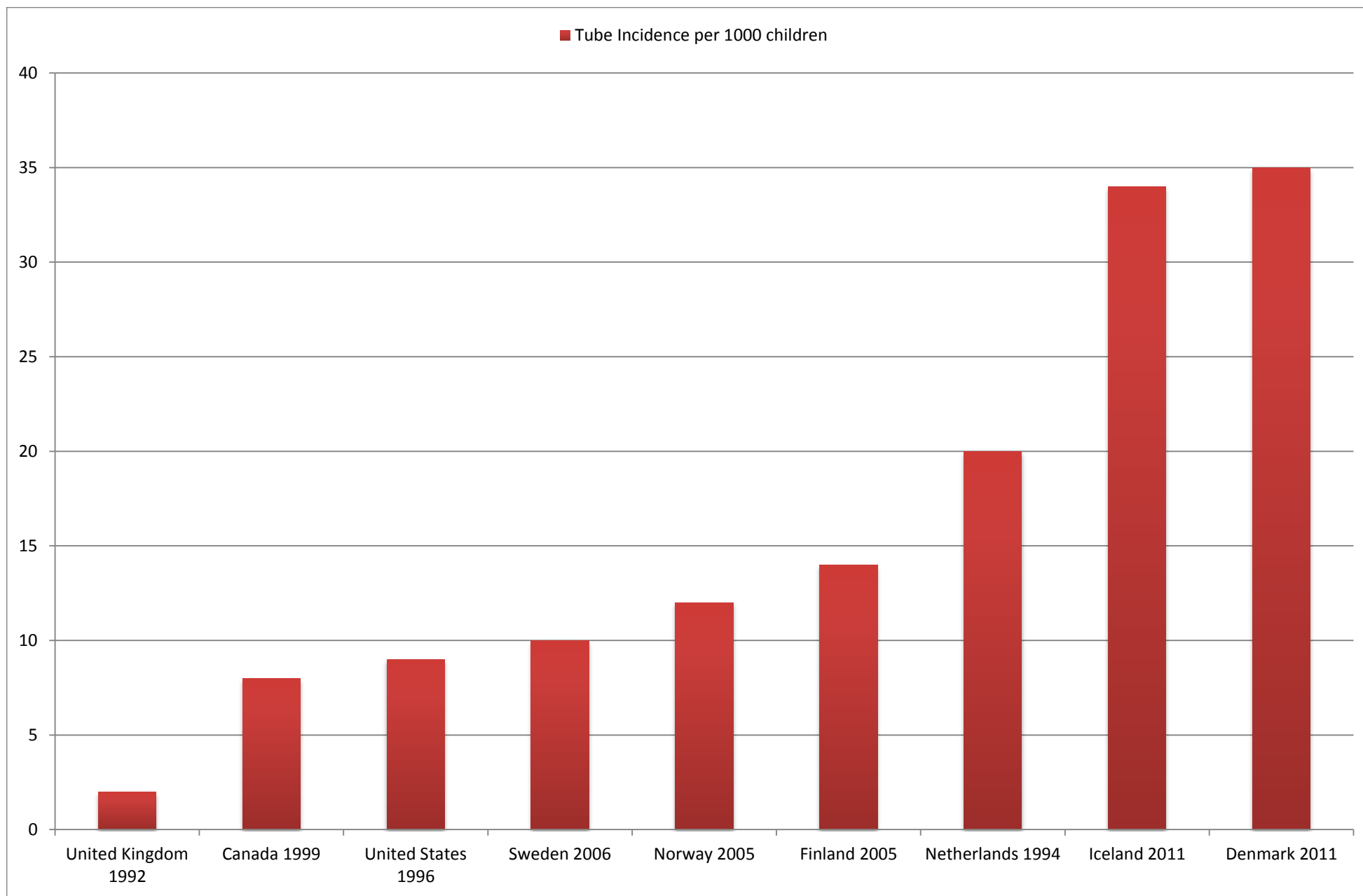

Supplement: S1 Fig — The overall incidence of ventilation tubes in Denmark of 35/1000 for children 0–15 years of age compared to incidences in other developed countries published in the literature [10–15,28,29]. (PDF) [file pone.0165657.s001.pdf]

**Fig.S2: Causality diagram**

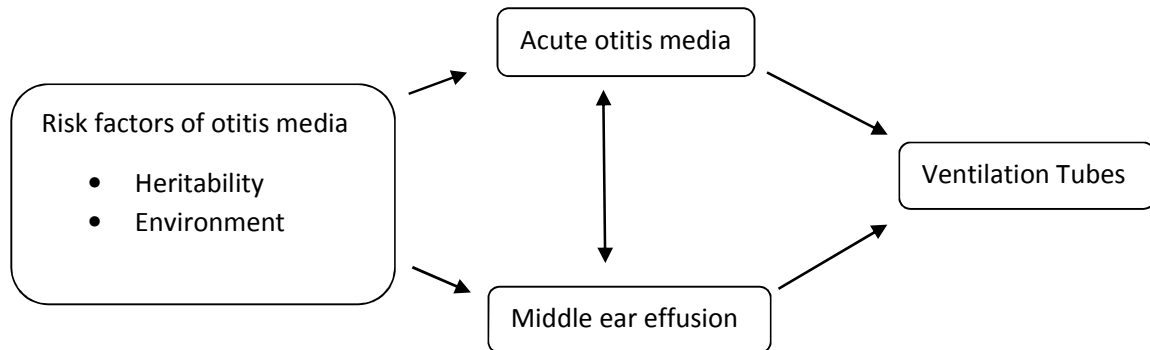

Supplement: S2 Fig — (PDF) [file pone.0165657.s002.pdf]
